# Variable spikes in tick-borne encephalitis incidence in 2006 independent of variable tick abundance but related to weather

**DOI:** 10.1186/1756-3305-1-44

**Published:** 2008-12-09

**Authors:** Sarah E Randolph, Loreta Asokliene, Tatjana Avsic-Zupanc, Antra Bormane, Caroline Burri, Lise Gern, Irina Golovljova, Zdenek Hubalek, Natasa Knap, Maceij Kondrusik, Anne Kupca, Milan Pejcoch, Veera Vasilenko, Milda Žygutiene

**Affiliations:** 1 Department of Zoology, University of Oxford, South Parks Road, Oxford, OX1 3PS, UK; 2 Centre for Communicable Diseases Prevention and Control, Vilnius, Lithuania; 3 Ministry of Health of the Republic of Lithuania, Vilnius, Lithuania; 4 Institute of Microbiology and Immunology, Faculty of Medicine, Ljubljana, Slovenia; 5 State Agency "Public Health Agency", Riga, Latvia; 6 Institut de Parasitologie, Université de Neuchâtel, Switzerland; 7 National Institute for Health Development, Tallinn, Estonia; 8 Institute of Vertebrate Biology, Academy of Sciences, Brno, Czech Republic; 9 Department of Infectious Diseases, Medical Academy, Bialystok, Poland; 10 Department of Comparative Tropical Medicine and Parasitology, Ludwig Maximilian University, Munich, Germany

## Abstract

**Background:**

The incidence of tick-borne encephalitis showed a dramatic spike in several countries in Europe in 2006, a year that was unusually cold in winter but unusually warm and dry in summer and autumn. In this study we examine the possible causes of the sudden increase in disease: more abundant infected ticks and/or increased exposure due to human behaviour, both in response to the weather.

**Methods:**

For eight countries across Europe, field data on tick abundance for 2005–2007, collected monthly from a total of 41 sites, were analysed in relation to total annual and seasonal TBE incidence and temperature and rainfall conditions.

**Results:**

The weather in 2006–2007 was exceptional compared with the previous two decades, but neither the very cold start to 2006, nor the very hot period from summer 2006 to late spring 2007 had any consistent impact on tick abundance. Nor was the TBE spike in 2006 related to changes in tick abundance. Countries varied in the degree of TBE spike despite similar weather patterns, and also in the degree to which seasonal variation in TBE incidence matched seasonal tick activity.

**Conclusion:**

The data suggest that the TBE spike was not due to weather-induced variation in tick population dynamics. An alternative explanation, supported by qualitative reports and some data, involves human behavioural responses to weather favourable for outdoor recreational activities, including wild mushroom and berry harvest, differentially influenced by national cultural practices and economic constraints.

## Background

The epidemiology of tick-borne encephalitis (TBE) in Europe is characterized by marked variability in space and time on both large and small scales. One recent event currently generating much speculation is the dramatic spike in incidence that occurred in 2006 in several countries: in Switzerland, Germany, Slovenia and Czechland (i.e. Czech Republic), incidence exceeded average levels for the previous decade by 79–183%, and was markedly higher than for any previous single year (Table 1). In Poland, Lithuania, Slovakia, Italy and France, TBE incidence was also high, but did not exceed that seen in some other recent years. In 2007, incidence reverted to average or below average levels in all these countries, while in Sweden and Norway a steady upward trend continued. In Estonia, Latvia, Finland and Hungary, there was very little change over the past three years, with incidence lower than average for the past decade.

**Table 1:** Annual TBE cases 2005–07, compared with means over the previous decade.

	Annual TBE cases			
	1995–04 mean ± 1 st dev	2005	2006	2007
Switzerland	92 ± 29	208	245	111
Germany	193 ± 72	432	546	238
Slovenia	231 ± 81	297	445	196
Czechland	574 ± 115	643	1029	546
Poland	214 ± 71	174	316	233
Lithuania	416 ± 194	242	462	234
Slovakia	75 ± 15	50	91	46
Italy	14 ± 9	19	30	17
France	3 ± 3	0	6	0
Sweden	94 ± 39	130	163	190
Norway	2 ± 1	0	5	12
Estonia	233 ± 98	164	171	140
Latvia	593 ± 388	142	170	171
Finland	24 ± 11	17	18	20
Hungary	117 ± 71	52	56	62

So far, the best explanation for this TBE spike in Czechland has centred around the unusual weather conditions of 2006 (described below), that are suggested to have improved tick survival over winter, accelerated the increase in spring questing activity by ticks, and encouraged more recreational activity by humans in tick-infested forests, particularly as conditions were especially favourable for good mushroom crops [1,2]. In some respects, this seems intuitively plausible. *Ixodes ricinus *ticks that transmit the TBE virus inhabit forests, where each life stage (larva, nymph, adult) spends one period of a few days feeding on a vertebrate host from a wide range of species; after each meal they spend seasonally variable periods of about 3–12 months in the leaf litter developing to the next stage, and then up to about two months on the vegetation questing for their next host [3,4]. The rate of development is temperature-dependent, and all ticks are highly sensitive to moisture stress [5-7]. Meanwhile, people obviously adjust their opportunistic recreational activities according to the weather. On the other hand, ticks of this species are highly cold-adapted, as witnessed by their distribution through northern Europe as far north as c.65°N in Sweden and northern Russia as far east as the Ural mountains, with no evidence that they actually suffer lower natural mortality rates leading to higher TBE incidence in warmer winter conditions [2]; the TBE spike actually followed an exceptionally cold winter (see below). Indeed, repeated freeze-thaw may be more harmful than persistent sub-zero temperatures, but there is no statistically significant relationship between the incidence of TBE or Lyme borreliosis over 1998–2004 and the number of days of thaw during the previous winter in western Czechland [2]. Ticks also undergo diapause over winter [8] with no development and only very occasional questing activity at temperatures below about 7°C [4,9-13], thereby minimizing the biological significance of increases in temperatures below this threshold level.

Here we test the impact of the variable weather conditions of 2005–07 on a) the timing of tick seasonal activity, b) the abundance of ticks, c) the seasonal distribution of TBE cases and d) the occurrence of a spike in TBE incidence in 2006, amongst eight European countries: Switzerland, Germany, Slovenia, Czechland, Poland, Lithuania, Estonia and Latvia.

## Methods and data

Data on monthly cases of TBE were acquired from national public health agencies or their web sites. TBE is a notifiable disease in each country considered.

**Figure 1:**
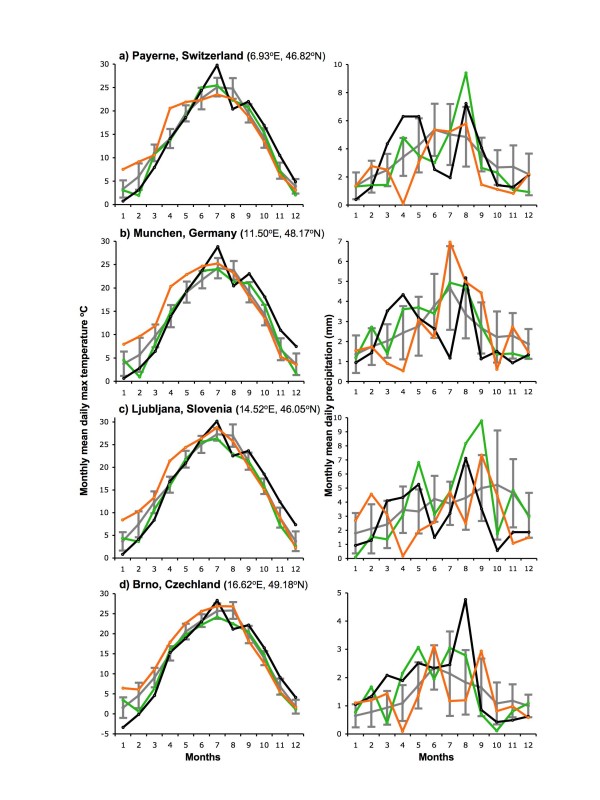
**Monthly means of daily maximum temperature (left column) and daily precipitation (right column) in 1989–07 at four locations across Europe**. Conditions in 2005 (green), 2006 (black) and 2007 (gold) are shown relative to means ± 1 st dev for the whole period 1989–07 (grey). Payerne, Munchen and Brno are close to tick sampling sites within particular parts of these countries. Ljubljana is taken as representative of each country across which tick sampling sites were scattered.

**Figure 2:**
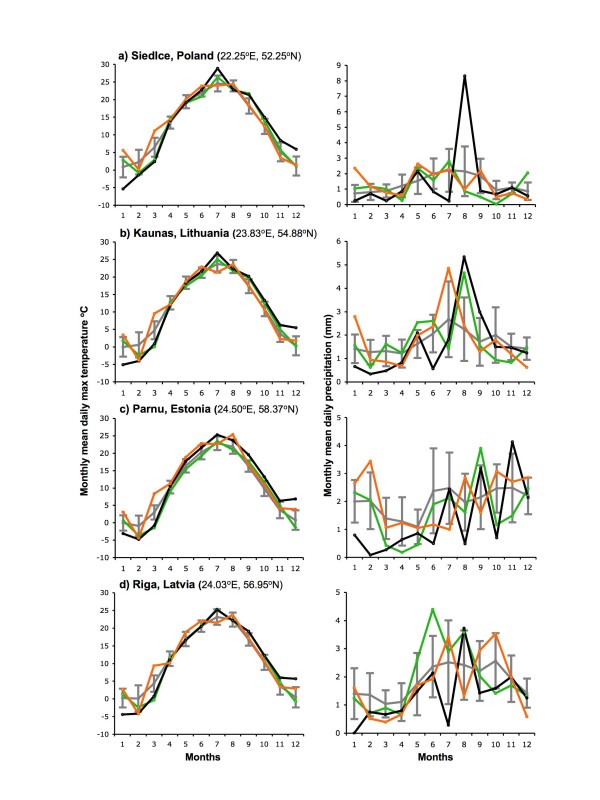
**Monthly means of daily maximum temperature (left column) and daily precipitation (right column) in 1989–07 at four locations across Europe**. Conditions in 2005 (green), 2006 (black) and 2007 (gold) are shown relative to means ± 1 st dev for the whole period 1989–07 (grey). Siedlce is close to tick sampling sites within NE Poland. Kaunas, Parnu and Riga are taken as representative of each country across which tick sampling sites were scattered.

**Figure 3:**
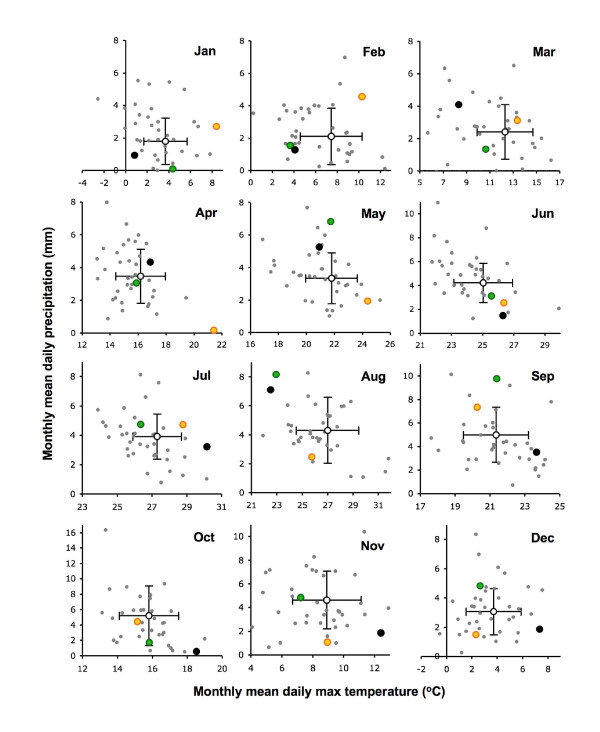
**Scattergrams of monthly mean daily precipitation and daily maximum temperature for each year for Ljubljana, Slovenia**. 1970–2004 (grey dots), 2005 (green), 2006 (black) and 2007 (gold). Mean ± 1 st dev for the period 1989–2007 (open circle and bars).

**Figure 4:**
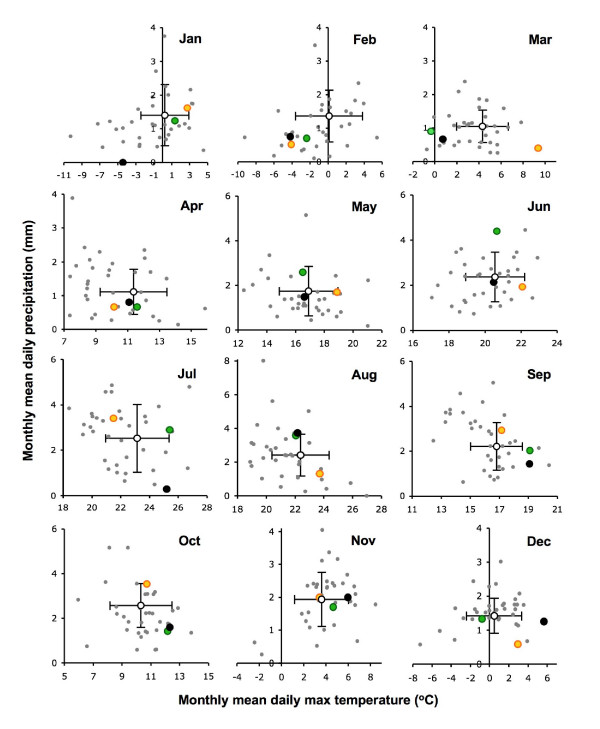
**Scattergrams of monthly mean daily precipitation and daily maximum temperature for each year for Riga, Latvia**. 1970–2004 (grey dots), 2005 (green), 2006 (black) and 2007 (gold). Mean ± 1 st dev for the period 1989–2007 (open circle and bars).

Daily maximum temperature (°C) and daily precipitation (mm) were downloaded from the European Climate Assessment web site [[14], available at ] for the years 1970–2007 for a representative site in each of eight countries (locations shown in Figures 1 and 2). Those for the site in Switzerland were provided by Professor Martine Rebetez (Swiss Federal Research Institute WSL, Lausanne). Given the very high degree of similarity in the weather patterns within any one country, and even between neighbouring countries, each site is taken as representative of the relative conditions in each year in each country, or, in larger countries, the part matched by tick sampling sites and TBE data. Over the course of a year, limiting conditions may possibly switch from minimum temperatures in the winter to maximum temperatures in the summer, but as these two daily measures are closely correlated the consistent use of maximum temperatures is adequate for inter-annual comparisons, which is the purpose of this study. Mean monthly values of each variable for each year were plotted against each other to give visual impressions of the temperature and moisture conditions for 2005, 2006 and 2007 relative to the full range of these conditions over 1970–2007 (Figures 3 and 4). As a significant step increase in temperatures occurred in 1989 throughout Europe [15], conditions for the past three years were compared statistically with the means (±1 standard deviation) for 1989–2007.

Questing ticks of all stages were counted monthly through 2005 (only in Slovenia), 2006 and 2007 at 4–8 sites per country, using 20 × 5 m standardized drags of 1 m^2 ^blankets (sites listed in Table 2). Ticks were sampled in specific parts of Switzerland (Bern), Germany (Bavaria), Czechland (NE and SE Moravia) and Poland (Podlaskie) and related to temporal variation in TBE incidence in the same part of the country. Tick sampling sites were scattered throughout the other four relatively small countries, so national TBE incidence data were used. There is, however, a fundamental epistemological gap between tick data, meteorological data and TBE case data because of the different spatial scales at which the causal processes of each operate and the data are recorded. Ticks respond to micro-climate, but macro-climate records give reasonable estimates of the gross seasonal and annual differences experienced by ticks and show high degrees of spatial correlation. Macro-climate records from sites close to, or within the geographical limits of, the tick sampling sites are therefore sufficient for this analysis. Tick abundance in any one place cannot be related to local TBE incidence because the place of infection is commonly not known and cases are recorded on a scale that encompasses different sites where tick densities vary. Furthermore, stochasticity and statistical non-significance arise from the small number of TBE cases on small spatial scales. Nevertheless, temporal trends in tick abundance (but not absolute abundance) monitored at a number of sites can be compared with trends in TBE incidence to test for consistent correlations.

**Table 2:** Characteristics of tick seasonal dynamics and abundance and TBE incidence.

Tick sampling sites			Nymphs peak month		Nymphs annual total		TBE cases	
	long	lat	2006	2007	2006	2007	2006	2007
**Switzerland**								
Belp, Bern	7.5	46.9	6	6	**403**	126	42	5
Trimstein, Bern	7.6	46.9	6	6	381*	90		
Kiesen, Bern	7.6	46.8	6	4	321*	300		
Thoune, Bern	7.6	46.7	6	6	52	**116**		

**Germany (Bavaria)**							188	109
Dachau, Bavaria	11.3	48.3	6	5	76	**132**	0	2
Munchen, Bavaria	11.6	48.2	5	4	38	36	1	1
Amberg, Bavaria	11.8	49.5	5	5	76	**177**	8	3
Rosenheim, Bavaria	12.1	47.9	5	5	51	**184**	7	1
Passau, Bavaria	13.3	48.7	6	5	273	**483**	5	2

**Slovenia**								
Črni kal, Koper	13.9	45.5	4	3	350	295	5	1
Osolnik, Ljubljana	14.3	46.1	6	5	**221**	173	120	60
Rakovnik, Ljubljana	14.4	46.1	6	5	338	326		
Štefanja gora, Ljubljana	14.5	46.3	6	5	**473**	326		
Kamniška Bistrica, Ljubljana	14.6	46.3	5	5	**327**	146		
Sodrazica, Ljubljana	14.7	45.8	4	5	219	**347**		
Mozirje, Celje	15.0	46.3	4	5	368	424	44	31

**Czech Republic**								
Krnov, Bruntál district	17.7	50.1	5	4	268	287	18	8
Vranovska, Znojmo district	15.8	48.9	4	5	375	362	7	6
Obora, Brno-mesto district	16.5	49.3	5	3	185	195	44	18
Valtice, Bøeclav district	16.8	48.8	5	3	**83**	61	3	2

**Poland**								
Kolno, Podlaskie	21.9	52.4	6	6	58	**87**	155	97
Grajewo-Ruda, Podlaskie	22.5	54.0	6	6	**186**	92		
Siemiatycze, Podlaskie	22.9	52.4	5	6	26	**41**		
Suwalki, Podlaskie	23.0	54.1	6	5	70	**91**		
Bialystok, Podlaskie	23.2	53.1	6	4	81	**122**		
Hajnowka, Podlaskie	23.9	54.7	6	4	**92**	54		

**Lithuania**								
Klaipeda, Klaipeda	21.1	55.8	5	5	145	128	5	3
Radviliskis, Siauliai	23.6	55.8	6	5	73	77	17	5
Kedainiai, Kedainiu	24.0	55.3	7	5	126	144	32	10
Utena, Utenos	25.5	55.6	6	4	34	28	4	2

**Estonia**								
Puhtu, Laanemaa	23.6	**58.6**	5	5	**636**	197	2	2
Are, Parnumaa	24.5	58.5	-	5	**163**	128	23	21
Kilingi-Nomme, Parnumaa	24.9	58.2	6	5	61	50		
Andineeme, Harjumaa	25.5	59.5	6	5	88	76	19	23

**Latvia**								
Vergale, Liepaja district	21.2	56.7	5	4	73	66	10	11
Blidene, Saldu district	22.8	56.6	7	5	**37**	20	1	1
Lapmezciems, Tukuma district	23.5	57.0	5	5	146	172	7	12
Tireli, Riga district	23.8	56.8	4	4	61	**182**	47	48
Mezaparks, Riga city	24.2	57.0	4	6	**62**	32		
Ozolnieki, Jelgava district	23.8	56.7	5	4	86	85	3	0
Kombull, Kraslava district	27.1	56.0	5	5	**20**	11	1	0

Bold, > 20% difference. Nymphal *Ixodes ricinus *sampled monthly in 2006 and 2007 at 41 sites in 8 countries. *Slight underestimates for 2006 because ticks initially (Mar-May) sampled at a site at 200 m higher altitude. TBE cases refer to numbers registered in each administrative region encompassing the tick sampling sites.

Blankets are relatively inefficient at picking up ticks and yield only approximate indices of true tick density. Comparisons of absolute tick densities in different places are inappropriate due to the differential sampling biases between operators and the small number, but high heterogeneity, of sites. Given standardized methods, however, blanket-dragging routes within tens of metres of each other throughout the 2–3 years and the equal efforts by the same operators throughout each year in this study, the indices can reveal crude comparisons of monthly and annual abundance from year to year. Here we use the measured indices of nymphal density to make inter-annual comparisons at each site of the timing and abundance of the tick stage that is sampled most reliably and also most likely to infect humans. Note that the total number of observations for each factor varies because not all sites gave unequivocal measures of each factor presented in the Results (if, for example, ticks were already active at the first sampling date in 2006, or showed equal high abundance over more than one month).

It is incorrect to look for formal correlations between the abundance of ticks and environmental factors, because such factors do not drive abundance but rather the rates of the underlying causal demographic processes. *I. ricinus *experiences rates of mortality and development from previous stages determined by a number of factors each acting at different times before the appearance of ticks in the questing population. The common perception that warmer weather in winter and spring will result in higher tick numbers and therefore higher TBE incidence is tested simply by comparing the relative measures of these variables associated with the TBE spike in 2006.

## Results

### Exceptional weather conditions 2006–2007

In the countries considered here, the salient features of the monthly means of daily maximum temperature and daily precipitation with respect to the present investigation (Figures 1, 2, 3 and 4) are as follows. In 2006, after an exceptionally cold (- > 1 st dev) period from January to March, each month from July to December was exceptionally warm (+ > 1 st dev) compared with the 1989–2007 average, and almost all were also drier than average, except for August that was atypically cool and wet. The unusually warm summer started as early as June in Switzerland, Bavaria (Germany) and Slovenia (Figure 3); it was least marked in Latvia (Figure 4), where only September and December were exceptionally warm, and the reversal in August was least marked in Latvia, Lithuania and NE Poland (wet but average temperatures), and Estonia (warm and dry).

Exceptionally warm conditions persisted from December 2006 through the first half of 2007, until as late as June in Bavaria, SE Czechland and Estonia, but this was least consistent in NE Poland, Lithuania and Latvia, where February was also particularly cold and spring was average. In all countries, parts of the summer and autumn were commonly much wetter in 2007 than 2006, but there was no consistent pattern.

The significant point is not that all these individual months were extreme relative to the past, but that there was an exceptional combination of far-from-average weather over a long period from mid 2006 to mid 2007 in most, but not all, of these eight countries.

### Timing of tick questing activity in relation to weather

**Figure 5:**
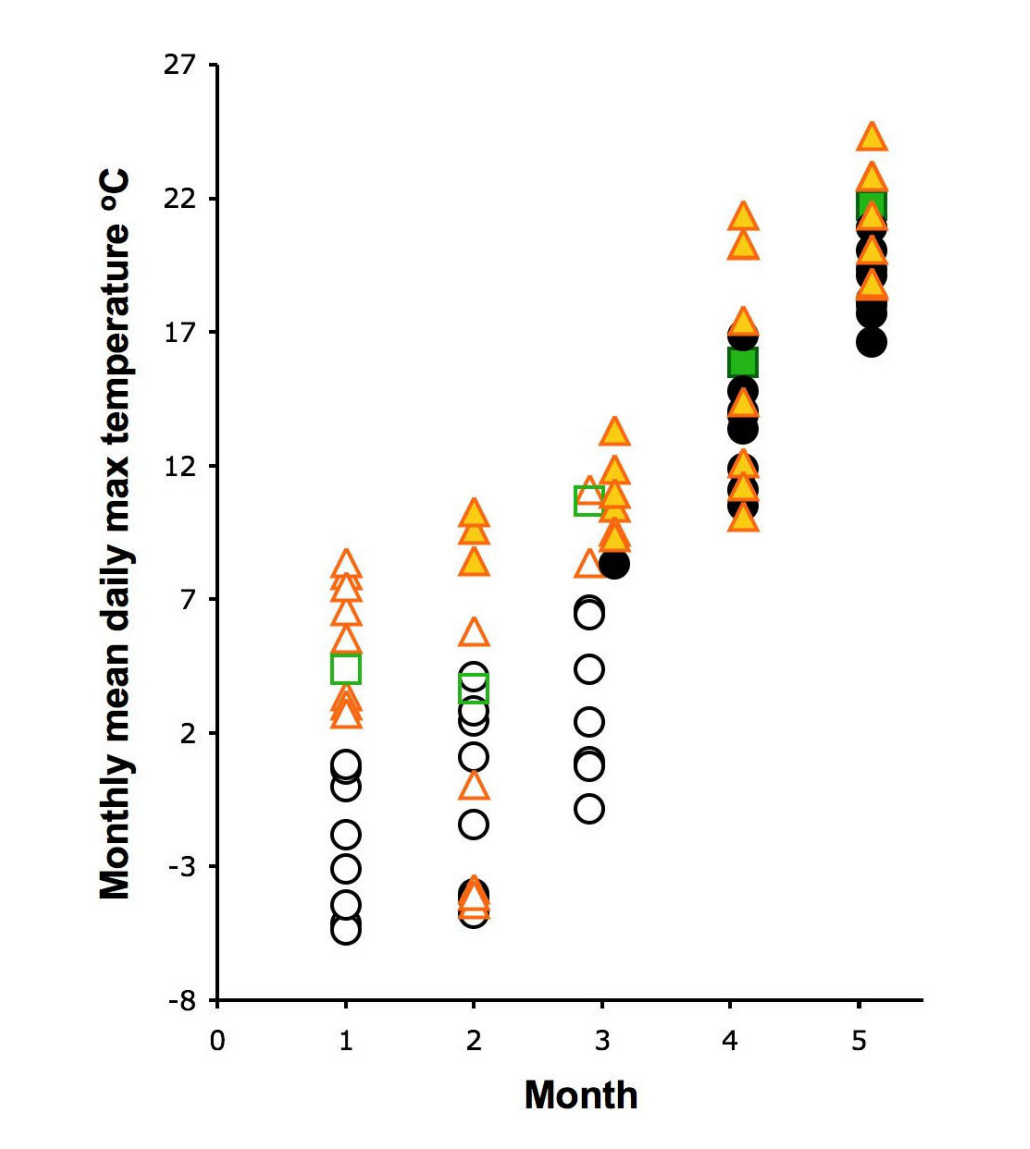
**Month of onset of activity by *Ixodes ricinus *nymphs in relation to monthly mean daily maximum temperature**. Averages for 4–7 tick-monitoring sites in each of Switzerland, Germany, Slovenia, Czechland, Poland, Lithuania, Estonia and Latvia. Temperature recorded at locations near to, or within the geographical limits of, tick sampling sites (see text and Figures 2 and 4 legends). Inactive ticks (open symbols) or active ticks (closed symbols) for 2005 (green square), 2006 (black circle) and 2007 (gold triangle).

**Figure 6:**
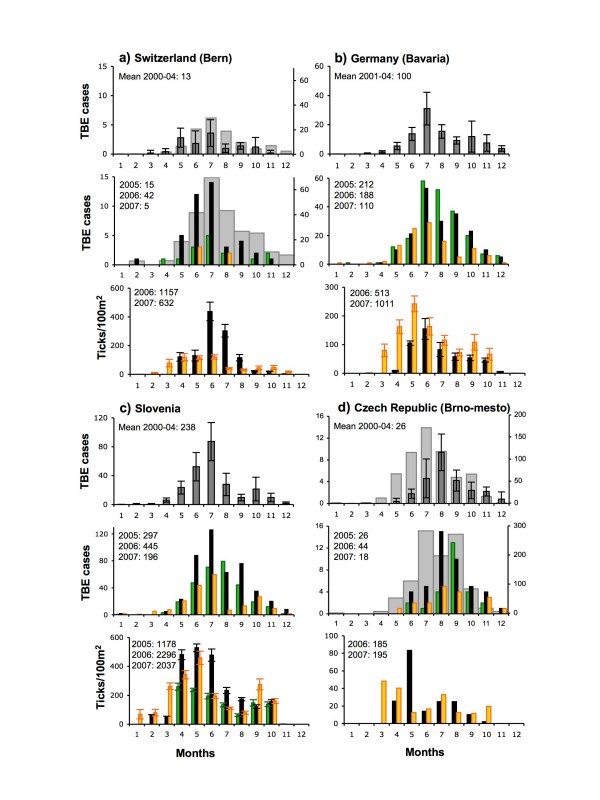
**Monthly distributions of cases of TBE and monthly densities of ticks**. a) Bern, Switzerland, b) Bavaria, SE Germany, c) Slovenia, d) Brno-mesto, SE Czechland. Upper and middle histograms for each country: means (± 1 st dev) over 2000–04 (grey), 2005 (green), 2006 (black) and 2007 (gold). In a) TBE case numbers for all Switzerland for 2000–04 (mean annual total 101) and for 2006 (total 259) are shown as pale grey bars and on the right-hand *y*-axis, behind the data for Bern; in d) TBE case numbers for all Czechland for 2000–04 (mean annual total 623) and for 2006 (total 1029) are shown in the same way. Lower histogram for each country, mean (± 1 st dev) monthly densities of ticks at 4–7 sampling sites matched to the above TBE incidence areas (locations given in Table 2) over 2005 (green), 2006 (black) and 2007 (gold). The tick data are advanced by one month in relation to the TBE data, to account for the approximate delay between tick bite and TBE registration. Annual total numbers of TBE cases and counted ticks for each year are shown.

**Figure 7:**
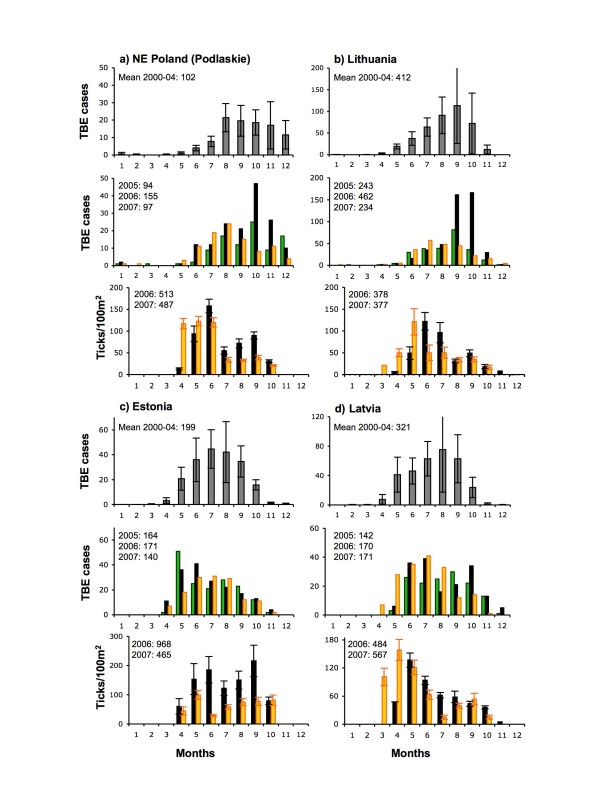
**Monthly distributions of cases of TBE and monthly densities of ticks**. a) Podlaskie, NE Poland, b) Lithuania, c) Estonia and d) Latvia. Upper and middle histograms for each country: means (± 1 st dev) over 2000–04 (grey), 2005 (green), 2006 (black) and 2007 (gold). Lower histogram for each country, mean (± 1 st dev) monthly densities of ticks at 4–7 sampling sites matched to the above TBE incidence areas (locations given in Table 2) over 2005 (green), 2006 (black) and 2007 (gold). The tick data are advanced by one month in relation to the TBE data, to account for the approximate delay between tick bite and TBE registration. Annual total numbers of TBE cases and counted ticks for each year are shown.

As expected from the temperature-dependence of tick activity, in 2007 questing nymphs were recorded up to 1–2 months earlier than in 2006 at 27 out of the 33 sites at which this could be reliably scored, generally appearing in large numbers in March or April (even in January at the site near the Adriatic coast of SW Slovenia) once the monthly mean daily maximum temperature had exceeded c.7°C (Figure 5). Correspondingly, the seasonal peak was reached 1–2 months earlier in 2007 (typically in May, but as early as March) at 22 out of 40 sites, in the same month at 13 sites and later at 5 sites (Table 2 and Figures 6 and 7). Then, from June or July onwards until the end of the summer, tick numbers were lower in 2007 than in 2006 (except in Bavaria), most likely because the questing tick population was depleted through natural mortality and as ticks found hosts earlier, and was not replenished by new recruits until the autumn (see discussion).

### Abundance of ticks in relation to weather

The abundance of questing ticks in any year is determined by mortality rates during development from the previous life stage over the past 3–12 months (i.e. usually including the winter period), contemporary weather conditions that determine tick activity, and also the density of wildlife hosts that remove ticks from the questing population. There is no evidence from simple comparisons of tick abundance in 2006 and 2007 from 41 sites in eight countries that the unusually warm conditions from July 2006 to June 2007 allowed better tick survival, greater activity levels and therefore greater abundance. Conversely, the very cold late winter of 2006 evidently did not adversely affect tick abundance. In 2007, the annual totals of monthly nymphal tick counts were lower (< 80% of 2006 levels) at 13 sites, higher (> 120% of 2006 levels) at 11 sites and differed by < 20% at the remaining sites (Table 2). Likewise, the seasonal peak numbers of nymphs were lower in 2007 at 16 sites, higher at 11 sites and < 20% different at the remaining sites (mean monthly densities per country shown in Figures 6 and 7 are influenced by certain sites where ticks were most abundant). Tick numbers were most consistently higher in 2007 in Bavaria (4 out of 5 sites), changed least in Lithuania (all 4 sites) and Czechland (4 out of 5 sites), and showed strongly inconsistent patterns elsewhere.

Likewise, data available only from Slovenia indicate that the much greater abundance of ticks in 2006 than in 2005 at all seven monitoring sites (Figure 6c) followed 18 months (January 2005 to June 2006) of temperature and rainfall conditions that were very close to the long-term average (Figures 1c and 3), apart from February and August 2005 and January-March 2006 that were exceptionally cold.

### Occurrence of TBE spike in 2006 in relation to weather

The spike in TBE incidence in 2006 in Switzerland (166% above the average for 1995–2004), Germany (183%), Slovenia (93%) and Czechland (79%) (Table 1) coincided with the extreme weather of June-December that year. On the other hand, in 2005 TBE incidence was also c.125% above average in both Switzerland (but not the canton of Bern) and Germany (including Bavaria) despite unexceptional weather. Likewise, in Poland (2006 incidence 48% above the 1995–2004 average) and Lithuania (11%) the incidence was as high or higher in several other years of the past decade (see ) in the absence of unusual weather patterns; although in the highest year, 2003, NE Poland experienced hot dry weather from May to September (including August) similar to 2006, Lithuania did not. In Latvia and Estonia, TBE incidence was no higher in 2006 than in other years, but while the 2006 summer-autumn weather was less extreme in Latvia, in Estonia it was as extreme as elsewhere except without the cool wet August. Thus it is clear that the association between exceptionally high annual TBE incidence and unusual weather patterns of the sort seen in 2006 is not consistent between countries, indicating that other factors act differentially in each country.

### Relationship between tick abundance and TBE incidence

As a major determinant of infection risk to humans (as distinct from human exposure to that risk) is the abundance of infected ticks, and as this is determined more by tick density than the relatively uniform infection prevalence of TBE virus that rarely exceeds 1%, TBE incidence might be expected to vary directly with tick abundance. This, however, is not the case. In the six countries that showed a TBE spike in 2006, markedly fewer (average 49 ± 25% fewer) TBE cases were recorded in 2007 than in 2006 in each of the regions where ticks were monitored, despite the higher or similar abundance of ticks at 22 out of the 30 sample sites (Table 2). In Estonia and Latvia, moreover, similar TBE incidences were recorded in each year despite markedly higher (27–223%) tick abundance in 2006 at 5 out of the 11 monitoring sites (similar abundance at 5 of the other sites). For reasons mentioned above (see Methods) relative tick densities recorded at the sample sites cannot be taken as representative of the relative risk of infection in each region, and are therefore not expected to be correlated with spatial variation in TBE incidence (as indeed they are not). Nevertheless, these broad inter-annual comparisons indicate that factors other than tick density determine temporal variation in human infections, and specifically the spike in 2006.

### Variable mis-matches between tick and TBE seasonality

**Figure 8:**
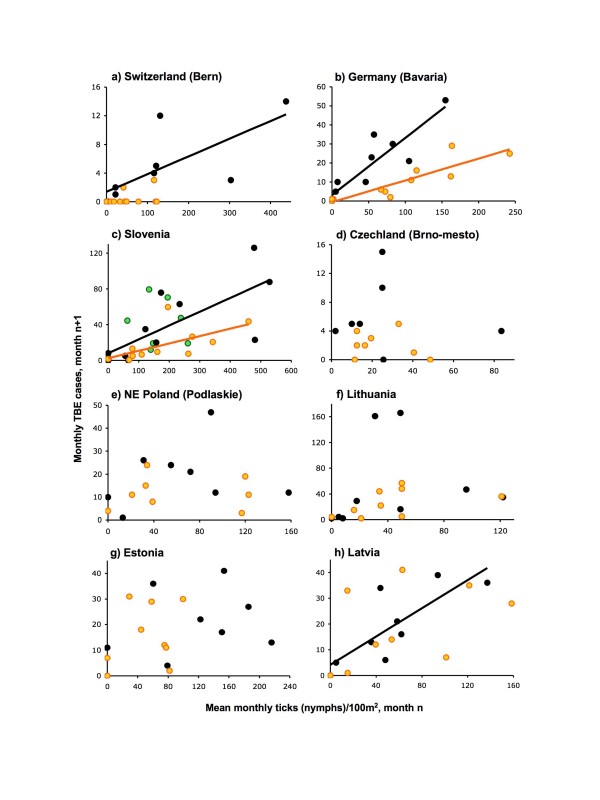
**Relationships between monthly TBE cases in month n+1 and mean tick abundance in month n for all tick sampling sites within the region from which TBE cases are counted, for 2005 (green), 2006 (black) and 2007 (gold)**. Only the following show statistically significant correlations: a) Bern, Switzerland 2006, *R*^2 ^= 0.531, n = 9, p < 0.05 (correlation between TBE and ticks in concurrent months is stronger, *R*^2 ^= 0.781, n = 10, p < 0.01); b) Bavaria, Germany 2006, *R*^2 ^= 0.820, n = 11, p < 0.001; 2007, *R*^2 ^= 0.784, n = 11, p < 0.001; c) Slovenia 2006, *R*^2 ^= 0.532, n = 11, p < 0.05; 2007, *R*^2 ^= 0.369, n = 11, p < 0.05; h) Latvia 2006, *R*^2 ^= 0.639, n = 9, p < 0.01.

Clues to interpreting the variable association between unusually high TBE incidence and exceptional weather, and the underlying causes, can be gleaned from examining variation in the degree to which seasonal patterns of TBE cases match those of tick abundance and whether this changed in 2006 (Figures 6, 7 and 8). Given the incubation period between infection and recognisable symptoms during the second phase of the biphasic illness (average 16–25 days, but sometimes much longer [16]), and the delay during diagnosis and reporting, the seasonal variation in TBE cases is expected to follow the seasonal variation in tick abundance by approximately one month (as shown by the offset x-axes in Figures 6 and 7). In Bern (Switzerland) over 2000–07, TBE was actually recorded in the month following the tick bite in 52% of 48 cases where the latter was known.

This expected match in seasonality is more or less met in Switzerland (Bern), Germany (Bavaria) and Slovenia (Figures 6a, b, c); TBE case numbers typically peak in June-August, decreasing during the autumn once ticks are less abundant. Compared with the baseline mean seasonal TBE pattern over 2000–04, and also the situation in 2007, the excess of TBE cases in 2006 (and to a lesser extent in 2005) in Bavaria, Switzerland and Slovenia started in June or July and continued to follow an elevated version of the typical seasonal curve until the end of the year. Disproportionate monthly increases occurred in August and September in Bavaria and Slovenia and in October in Switzerland as a whole (pale grey bars, Figure 6a), but not in Bern, even though seasonal tick numbers were relatively low by then. This is reflected in the typical, but not invariable, significant correlation between mean tick numbers counted in month n and TBE cases in month n+1 (Figures 8a, b, c), but the higher elevation of the slopes for 2006 again emphasize the spike in 2006 independent of greater tick abundance.

In SE Czechland (Brno-mesto district, Jihomoravský administrative region) (Figure 6d) and also the northeast (Bruntal district, Moravskoslezský region – data not shown), the seasonal peak in TBE incidence typically occurs in August, one month later than the mean for Czechland as a whole [1] (pale grey bars, Figure 6d) or for West Bohemia [17], despite the relatively early peak in tick abundance (March–May). In 2006, TBE cases were higher throughout the normal season, but there was a disproportionate increase in the autumn when ticks were at low levels. Monthly TBE incidence was not correlated with mean tick abundance in either 2006 or 2007 (Figure 8d). Curiously, in 2005 the seasonal peak shifted to September, with fewer cases than usual in July and August.

A similar mis-match between tick and TBE seasonality is also seen in Latvia, Lithuania and NE Poland, to increasing degrees in that order (Figures 7a, b and 7d and 8a, b and 8d), where large numbers of TBE cases occur much later in the year relative to peak tick numbers than in the above countries. In Lithuania and Poland, TBE incidence is particularly low in spring and early summer. The excess in TBE cases in 2006 was limited to, but very extreme in, September and October in Lithuania and October and November in NE Poland. Estonia showed an unusual pattern, particularly marked in 2006, in that although tick abundance remained high until the autumn, TBE incidence declined after the summer (Figure 7c) so again, there was no correlation between the two (Figure 8g).

### Human outdoor activities

**Figure 9:**
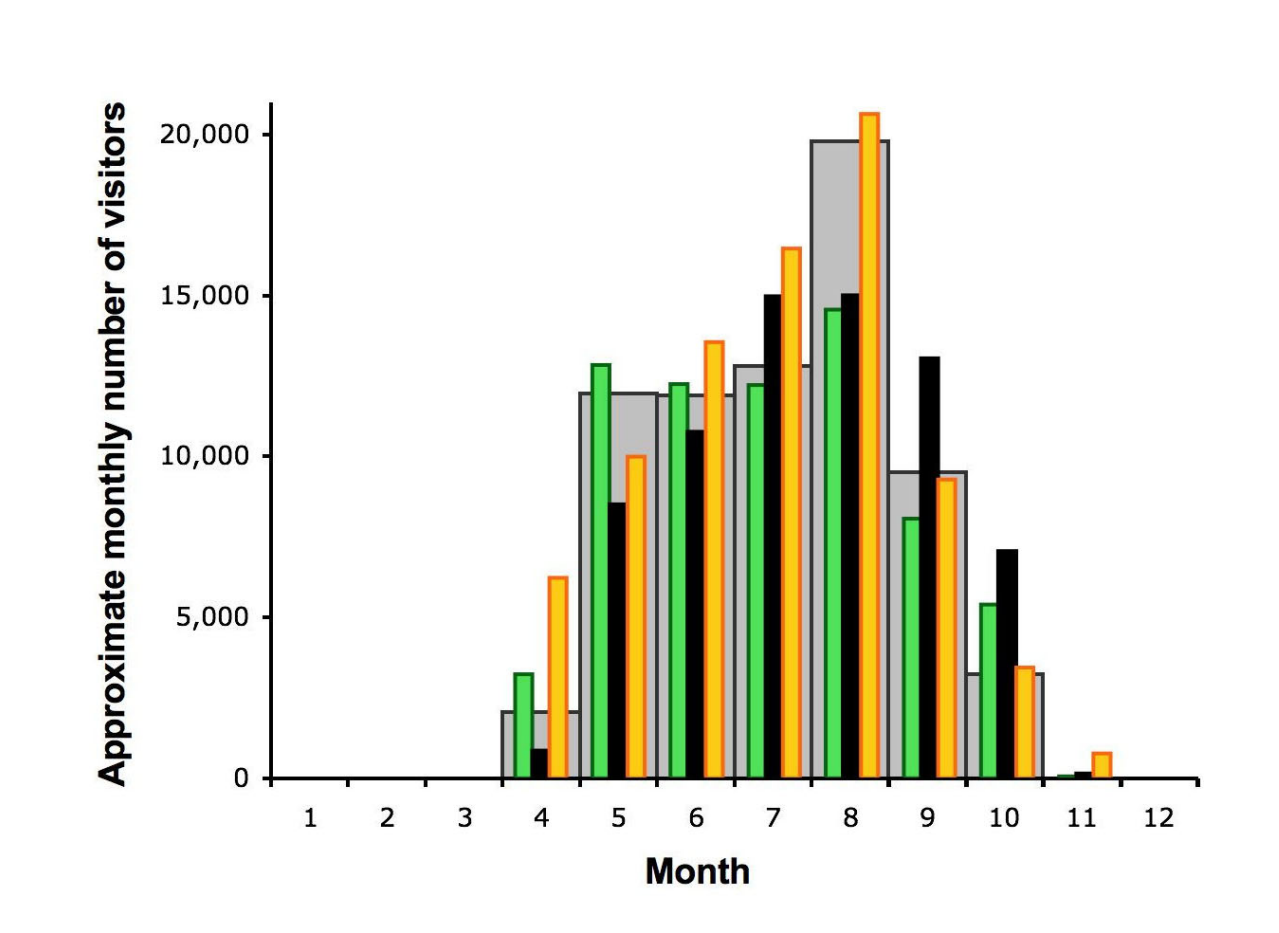
Relative numbers of monthly visitors for the years 2003–04 (grey), 2005 (green), 2006 (black) and 2007 (gold) to the Logarska dolina national park in the Logar valley of Slovenia, close to the tick sampling site at Mozirje.

Data on seasonal and annual variation in numbers of visitors to the Logarska dolina national park in the Logar valley of Slovenia provide some indication of the increase in outdoor recreational activities in response to the clement weather in the second half of 2006. This park, close to the tick sampling site at Mozirje in the north of Slovenia (Table 2), is one of the larger parks in the country in which the timing and relative volume of human activity reflects that in the country as a whole. Relative numbers of monthly visitors for 2003–07 (Figure 9) were monitored by collection of entrance fees daily between 08.00 and 18.00 hrs from April to November. This underestimates total visitor numbers by not fully recording those who arrive on foot, cycle or public transport, and motorcyclists with season tickets or who do not use the main entrance. Nevertheless, it is clear from this index that there were more visitors than average during the unusually warm weather in July, September and October 2006, and in April (but not May) and July 2007, with fewer visitors during the cool weather in August in both 2005 and 2006 (Figure 1c).

## Discussion

### Impact of weather on (seasonal) tick abundance

There is no doubt that ticks are highly sensitive to abiotic conditions, with development rates driven by temperature and mortality rates determined principally by moisture stress. It is these spatially variable factors operating consistently over long periods that apparently set the limits to the focal distribution of TBE across Europe [18,19]. Nevertheless, that does not mean that annual variation in the weather, well within the existing range of both ticks and TBE, necessarily results in significant contemporary changes in tick abundance. The data presented here reveal that unusually high temperatures from June or July of 2006 through to May or June of 2007, sometimes accompanied by lower than average rainfall, did not result in an increase in tick abundance in 2007 at 30 out of the 41 sites, while increased tick numbers from 2005 to 2006 at all 7 sites in Slovenia followed an exceptionally cold winter in the middle of more than a year of average weather conditions. The earlier onset of tick activity in 2007, as temperatures exceeded the threshold level (c.7°C) one or two months earlier, merely resulted in an earlier decline after the peak, usually to lower levels from July to October in 2007 than in 2006. This is in accord with existing understanding of tick life cycle dynamics, that new cohorts of each unfed tick life stage are recruited each autumn when development from the previous stage is completed more or less synchronously irrespective of the time of feeding; the majority then enter diapause over winter and emerge the following spring [11]. Only in Estonia and NE Poland were (presumed) newly recruited nymphs more abundant in the autumn of 2006 than 2007. If this was due to faster development from engorged larvae during the warm summer of 2006, it was not an effect evident everywhere.

Clearly, although warmer temperatures may shift the seasonal pattern of tick questing activity through direct behavioural responses (Figure 5) or accelerated development rates [20], there is no simple consistent association between warmer weather and higher tick abundance as has been claimed [21,22]. In the absence of the ideal long-term monitoring of all tick life stages and their hosts in a wide variety of places, a tick population model would help to determine precisely which abiotic factors drive the variable abundance from year to year. This is distinct from the short-term response of ticks to moisture stress that temporarily inhibits their questing activity [7,10,23]. Interestingly, rainfall in summer and autumn of 2006 was below average in many months (apart from August), which, if anything, would have decreased tick activity, just at the time when the increase in TBE incidence occurred.

### Variable impact of weather on TBE incidence

The variable degree of mis-match between the tick and TBE seasonality in all years shown here might simply reflect the differential delay by each national or local public health service in registering TBE infections. Alternatively it indicates that even at their autumnal low levels, ticks are sufficiently abundant in nature to pose a significant risk of infection with TBE virus, and that the seasonality of TBE is driven by factors other than tick abundance. The two most obvious of these are variable prevalence of TBE virus infection in ticks that would alter the level of risk from the environment, and human behaviour that brings people into contact with ticks and determines the realised infection incidence in humans. Infection prevalence in questing nymphal ticks is generally very low, up to c.2%, and in any one year is determined by the degree of virus transmission from infected nymphs to infectible larvae while co-feeding on rodent hosts [24], which occurs principally during the preceding year or the spring and early summer of the year in question [11]. The force of this transmission route is positively related to the degree of synchrony in feeding by larvae and nymphs, in turn determined by the rate at which temperatures rise in the spring [18,19,25]. Temperature records indicate that spring temperatures may have been unusually favourable for TBE virus transmission in 2004–06 in Switzerland, Germany and Czechland (detailed analysis to be published separately), but not in any of the other countries considered here. Investigation of any resulting higher infection prevalence in ticks is under weigh, particularly with respect to any seasonal patterns sufficient to offset the low tick abundance in the autumn that could account for the excess TBE cases in that part of 2006.

Data on numbers of visitors to a national park in Slovenia, although limited, support the speculation that, not surprisingly, human recreational behaviour changed in response to the unusual weather of 2006 in a way that could have increased the contact between people and ticks. Furthermore, there was no consistent direct relationship between tick abundance and the TBE spike in 2006, on either an annual or seasonal basis, as much of the excess incidence occurred in the autumn after the seasonal decline in tick activity. This suggests that this spike could also have been driven by changes in human activities rather than in tick biology. In Czechland, there were many media reports of increased hiking and mushroom harvesting in 2006 at the expense of other outdoor activities, continuing to early November [1,2].

Taken together, all the data presented here indicate that variation in the weather has a marked impact on TBE incidence, not by affecting the abundance of ticks, but possibly by enhancing infection prevalence in ticks (no supporting data yet) and most likely by altering human activities (supported by some data). Why, though, was there a much more pronounced TBE spike in Switzerland, Germany, Slovenia and Czechland than in NE Poland or Lithuania, and no spike in Estonia (and Latvia), given the similar pattern of remarkably warm weather from mid-2006 to mid-2007, accompanied by below average rainfall for much of the summer and autumn of 2006, in all these eight countries except Latvia?

The answer may lie in the precise pattern of the spring temperature increase and therefore the virus transmission potential (but this does not apply to Slovenia), or in the purpose of people's outdoor activities in each country. In Switzerland, Germany, Slovenia and Czechland, apart from forest workers, most people visit forests for recreation, which would be expected to increase opportunistically in response to fine weather, as indeed the data for Slovenia support. This is the explanation offered for the high number of tick bites reported in the Neuchâtel region of Switzerland in June 2003, and ongoing tick bites during the autumn of 2004 and 2005 after the tick population was declining [26]. Furthermore, peak numbers of tick bites reported to the Public Health Agency in Riga, Latvia were independent of the variable local tick activity, but coincided with warm dry weekends suitable for human recreation in forests following wet weather likely to have promoted the growth of mushrooms [Figure 5 in [27]]. When conditions also favour the late summer and autumnal growth of mushrooms (and forest berries), as would high rainfall in August, good crops of these wild foods would be harvestable throughout the warm dry autumn. The great emphasis placed on recreational mushroom picking in Czechland [1] would account for a later peak in TBE seasonality relative to ticks even in years with average weather. Evidence for an increase in these activities in 2006 right through to November is available for Czechland [1], and evidence that such activities increase the risk of exposure to ticks and therefore TBE virus is available from survey data for Latvia [27].

Mushroom gathering is also important in NE Poland and Lithuania, from where large quantities of wild edible fungi are exported to Western Europe [28]; activities upon which people depend for their livelihood are likely to vary less by drawing in opportunistic recreational foragers in good years, although TBE cases were disproportionately high in the autumn in 2006. Indeed, in years when the crop is poor, additional efforts may be needed to secure a good harvest. This may explain why the TBE spike in 2006 was no higher than that in some other years in NE Poland (2003) and Lithuania (2000, 2003 and 2004), when the excess cases also occurred in the autumn. During 2000–07, only 8% of annual TBE cases in NE Poland and 13% in Lithuania were recorded before July, and 38–48% before September, suggesting very little human activity in forests before the mushroom season.

Although Estonia and Latvia have traditions of local mushroom and berry use, they are only minor exporters [28]. In both countries, relatively large proportions of the populations post-independence were employed in agriculture or subsistence activities likely to bring them into contact with ticks in forests and rough land [29], although this has declined over recent years. Neither nation, however, is renowned for the sort of sportive recreational activities that might increase opportunistically in favourable weather. The weather in Latvia was, in any case, less extreme in 2006.

## Conclusion

The data presented here all highlight the importance of investigating both ends of the biological spectrum, human behaviour and virus transmission dynamics, when searching for epidemiological explanations. To date there are reasons to consider the potential importance of human activity, rather than tick activity, in response to an unusual combination of weather over the second half of 2006 as the driving cause for the unusually high annual incidence of TBE in that year. This conclusion could be tested by exploring for any country-specific changes in the socio-demographic profiles of human TBE cases. That is not to say that ticks do not respond to weather conditions, but not apparently in ways that can account for these recent major events of TBE epidemiology. The sampling methods, however, would not have detected whether the same number of ticks prolonged the duration of their daily questing, thereby increasing risk. Tick numbers were noticeably high in September 2007 in Bavaria (Germany), Slovenia and NE (but not SE) Czechland (data not shown), when rainfall was also very high in these places, but the causal link between tick numbers and rainfall at this time of the year is unknown.

There remain several puzzles apparent in the data for which this broad-brush analysis has not found answers. In parts of Switzerland and Germany, for example, TBE incidence was also high in 2005 when the weather resembled that of 2006 (but in a less extreme way) only in June and August to October. TBE incidence plummeted in 2007 despite favourable conditions (warm and dry) for outdoor activities in the spring of that year during the season of peak tick abundance. Had conditions prior to this become less favourable for TBE virus transmission between co-feeding ticks resulting in lower infection prevalence in ticks? Were people responding to publicity about the risk of TBE following the spike in 2006, increasing their vaccination or avoiding tick-infested forests, as apparently happened from 1999 after the highest known levels of TBE incidence in Latvia [27]? Finally, no explanation has yet been found for the varying abundance of ticks in each year.

## Competing interests

The authors declare that they have no competing interests.

## Authors' contributions

SER conceived the study, carried out all the data analysis and wrote the manuscript. All other authors supervised or carried out tick sampling, and contributed data on TBE epidemiology, and made suggestions for the final manuscript.
